# Development and validation of a nomogram based on LASSO regression for predicting early postoperative polyp recurrence in patients with chronic rhinosinusitis with nasal polyps

**DOI:** 10.3389/fsurg.2026.1806350

**Published:** 2026-07-14

**Authors:** Hanshuang Zhang, Huizhen Zheng, Siqi Wang, Shile Xu

**Affiliations:** 1 Department of Otorhinolaryngology Head and Neck Surgery, Wenzhou Central Hospital, Wenzhou, Zhejiang, China; 2 Department of Otorhinolaryngology, Wenzhou People’s Hospital, Wenzhou, Zhejiang, China

**Keywords:** chronic rhinosinusitis with nasal polyps, early postoperative recurrence, LASSO regression, nomogram, risk factors

## Abstract

**Background:**

Chronic rhinosinusitis with nasal polyps (CRSwNP) has a high early postoperative recurrence rate, and precise individualized prediction tools are currently lacking.

**Objective:**

To construct and validate a nomogram model based on commonly used clinical indicators to predict the risk of early postoperative polyp recurrence in patients with CRSwNP.

**Methods:**

A retrospective cohort study design was adopted. A total of 260 and 114 patients with CRSwNP who underwent endoscopic sinus surgery in our hospital from January 2021 to April 2025 were selected as the training cohort and internal validation cohort, respectively. The training cohort was divided into a recurrence group and a non-recurrence group according to recurrence status within 6 months. In addition, 242 patients from Wenzhou People's Hospital were included as the external validation cohort. Independent influencing factors were screened using LASSO regression and multivariate logistic regression, and a nomogram prediction model was constructed. The model performance was evaluated using receiver operating characteristic (ROC) curves, calibration curves, and decision curve analysis.

**Results:**

Asthma, stage 3 sinusitis, Lund-Mackay score, eosinophils (EOS), and immunoglobulin E (IgE) were independent risk factors for early postoperative recurrence (*P* < 0.05). The AUCs (95% CIs) of the training cohort, internal validation cohort, and external validation cohort were 0.923 (0.882–0.963), 0.881 (0.827–0.936), and 0.890 (0.841–0.939), respectively. The calibration curves and Hosmer–Lemeshow test showed that the model had good calibration, and decision curve analysis indicated that it provided clinical net benefit.

**Conclusion:**

The nomogram model has certain predictive performance, can identify patients at high risk of early postoperative polyp recurrence in CRSwNP, and has the potential to serve as an important auxiliary tool for individualized postoperative clinical management.

## 1 Introduction

Chronic rhinosinusitis with nasal polyps (CRSwNP) occurs in the nasal cavity or nasal mucosa and is a common chronic inflammatory disease characterized by a long disease course and a high likelihood of multiple complications (1–3). The clinical manifestations of CRSwNP include nasal obstruction, headache, and olfactory dysfunction. Failure to achieve timely diagnosis and treatment may aggravate respiratory tract infections, reduce patients' quality of life, and in severe cases lead to meningitis or even death (4). Endoscopic sinus surgery can be considered the first-line treatment for CRSwNP, with the aim of restoring sinus ventilation and mucociliary clearance (5). However, CRSwNP is a chronic disease, and persistence after surgical treatment is possible; therefore, symptoms and polyps may recur at any time (6). Postoperative polyp recurrence in CRSwNP is associated with multiple risk factors, such as age, bronchial asthma, and allergic rhinitis (7). Currently, clinicians mainly rely on empirical judgment to assess the risk of recurrence. Although commonly used single indicators, such as the Lund-Mackay score or peripheral blood eosinophils (EOS), may provide certain predictive information, they are difficult to comprehensively reflect the complex pathophysiological process and cannot achieve precise individualized risk stratification (6, 7). In addition, existing prediction models often include numerous and complex variables, and practical tools constructed based on simple and readily available clinical indicators and externally validated are still lacking. This study aimed to identify the independent influencing factors for early postoperative recurrence in patients with CRSwNP through rigorous statistical modeling and to establish a prediction model capable of accurately identifying high-risk patients, thereby providing a scientific basis for implementing individualized postoperative management strategies. Compared with previous studies, the innovation of this study is mainly reflected in the optimization of the variable selection method, namely the use of LASSO regression for variable selection to overcome multicollinearity; the intuitiveness and practicality of the model, namely the construction of a visual scoring system, that is, a nomogram model; and the completeness of the validation system, as internal cross-validation was performed and external hospital data were also included for external validation.

## 2 Materials and methods

### 2.1 Materials

A total of 374 patients with CRSwNP who underwent surgical treatment at our hospital between January 2021 and April 2025 were selected as the study population. Using random allocation at a ratio of 7:3, the patients were divided into a training cohort (for Nomogram model construction) comprising 260 cases and an internal validation cohort (for preliminary validation) comprising 114 cases. In addition, 242 patients with CRSwNP from Wenzhou People's Hospital during the period from January 2021 to April 2025 were included as an external validation cohort. The same diagnostic criteria, inclusion and exclusion criteria, follow-up procedures, and definition of early postoperative recurrence were applied to the external validation cohort. The external validation cohort was used only for independent model validation and was not involved in variable selection or model development. All data from the external validation cohort were de-identified before analysis. The numbers of early postoperative recurrence cases in the training cohort, internal validation cohort, and external validation cohort were 52, 22, and 46, respectively. The baseline characteristics of the three cohorts are presented as raw distributions in Supplementary Table S1.

**Figure 1:**
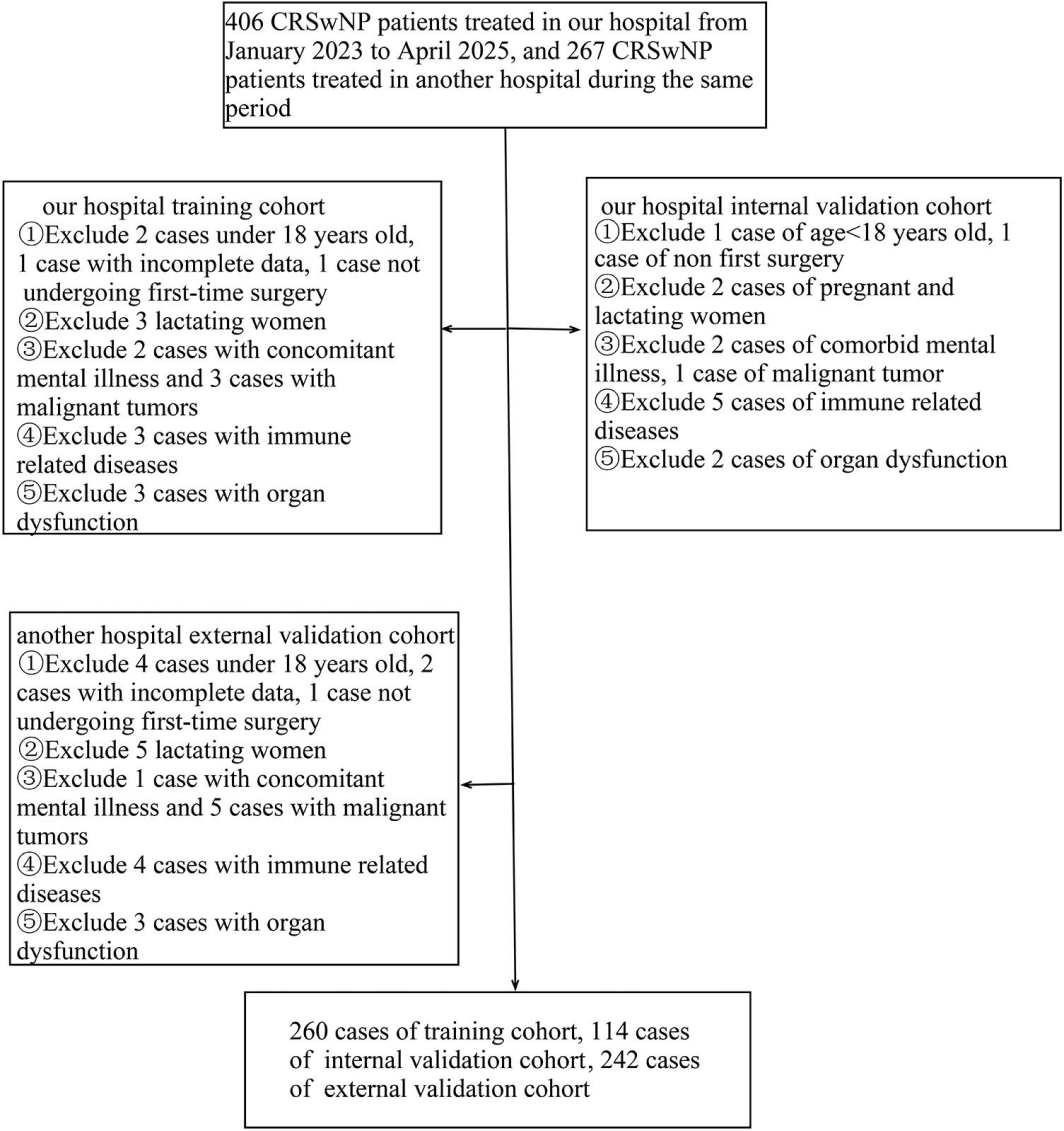
Case flow chart.

The inclusion criteria were as follows: ① meeting the diagnostic criteria for CRSwNP (8), with nasal endoscopy showing the osteomeatal complex with or without inflammatory lesions of the sinus mucosa, and all included patients were classified as type II; ② age ≥18 years, with complete clinical data and follow-up records; and ③ first-time surgical treatment. The exclusion criteria were as follows: ① pregnant or lactating women; ② patients with neuropsychiatric disorders or malignant tumors; ③ patients with immune-related diseases; and ④ patients with severe renal, hepatic, pulmonary, or cardiac dysfunction. The case collection flowchart is shown in Figure 1.

### 2.2 Methods

#### 2.2.1 Data collection

This study adopted a retrospective cohort design. Patients' clinical information was collected through the hospital electronic health record system, including: (1) preoperative baseline data: data collected from admission to within 24 h before surgery, including age, sex, smoking status (previous or current smoking history, defined as smoking at least one cigarette per day for more than 6 months), alcohol consumption, body mass index (BMI), disease duration, comorbidities (hypertension, diabetes mellitus, allergic rhinitis, and asthma), preoperative laboratory examinations [C-reactive protein (CRP), white blood cell count (WBC), platelet count (PLT), EOS, eosinophil cationic protein (ECP), immunoglobulin E (IgE), interleukin-1β (IL-1β), and interleukin-5 (IL-5)], sinusitis staging (9) (stage 1: nasal polyps with involvement of one group of paranasal sinuses; stage 2: nasal polyps with involvement of two groups of paranasal sinuses; stage 3: nasal polyps with involvement of all groups of paranasal sinuses, often accompanied by extensive involvement of the middle turbinate, cribriform plate, frontal sinus, and other structures), and Lund-Mackay score [according to the scoring system proposed by Lund and Mackay (10), which was assessed on preoperative sinus CT images by a radiologist blinded to patient outcomes. Six regions on each side, including the frontal sinus, maxillary sinus, anterior ethmoid sinus, posterior ethmoid sinus, sphenoid sinus, and ostiomeatal complex, were scored as no abnormality (0 points), partial opacification (1 point), or complete opacification/obstruction (2 points), with a total score ranging from 0 to 24]. Long-term use of nasal decongestants was defined as continuous preoperative use of *α*-adrenergic receptor agonist nasal sprays, such as oxymetazoline hydrochloride or naphazoline, for more than 7 days. (2) Perioperative data: Data on the day of surgery and during postoperative hospitalization were collected, including the number of polyps (single polyp was defined as a polyp confined to a single sinus or an isolated polyp visible only in one nasal cavity; multiple polyps were defined as polyps involving multiple sinuses or widely distributed in both nasal cavities), nasal septal deviation, middle turbinate resection, postoperative infection, postoperative nasal adhesion (defined as an abnormal bridge-like connection within the nasal cavity confirmed by nasal endoscopy during postoperative follow-up, commonly occurring between the inferior turbinate and nasal septum or between the middle turbinate and lateral nasal wall, resulting in narrowing of the airway), and postoperative nasal packing.

#### 2.2.2 Surgical procedures

All patients underwent nasal endoscopic examination and were treated strictly according to the standardized Messerklinger surgical approach. Before surgery, all patients received standardized medical treatment for 2 weeks, including nasal irrigation with normal saline and intranasal glucocorticoid spray. In patients with concomitant asthma or diabetes mellitus, the underlying diseases were required to be controlled to a stable state before surgery.All procedures were performed under general anesthesia with controlled hypotension. The uncinate process was first removed, and the anterior ethmoid sinus was adequately opened. Subsequently, the extent of sinus opening was determined according to the preoperative Lund-Mackay CT score and nasal polyp grading based on the Lund-Kennedy score. For patients with stenosis of the natural ostium of the maxillary sinus, the ostium was enlarged to a diameter of more than 5 mm. For patients with anatomical variations of the frontal recess, the frontal sinus drainage pathway was precisely opened, and lesions in the posterior ethmoid sinus and sphenoid sinus were routinely removed. During the operation, complete removal of polyps was emphasized while preserving normal sinonasal mucosa as much as possible.Nasal packing was removed on the second postoperative day, and a standardized postoperative care protocol was initiated immediately. All patients received continuous nasal irrigation with normal saline for 3 months. From the third postoperative day, regular long-term intranasal glucocorticoid spray was administered for 6 months. For patients with a preoperative eosinophil percentage ≥5%, short-term sequential oral glucocorticoid therapy was additionally given. The remaining patients received only routine intravenous antibiotics for infection prevention for 5–7 days. Nasal crusts and pseudomembranes were regularly removed once weekly within 2 weeks after surgery to ensure epithelialization of the surgical cavity.

#### 2.2.3 Assessment of postoperative recurrence

The definition of postoperative nasal polyp recurrence was strictly based on the criteria described in the European Position Paper on Rhinosinusitis and Nasal Polyps 2020 (EPOS 2020) formulated by Fokkens et al. (8).After discharge, patients with CRSwNP were followed up for 6 months through a combination of WeChat communication, telephone calls, and voluntary outpatient visits. During follow-up, if symptoms such as nasal obstruction, rhinorrhea, hyposmia, or a sensation of a foreign body in the nose persisted for 1 month without relief, and computed tomography (CT) examination showed soft tissue shadows in the nasal cavity or paranasal sinuses, increased sinus density, and mucosal thickening, and newly formed polyps or polypoid changes of the mucosa in the original surgical cavity were confirmed by nasal endoscopy by two or more experienced otolaryngologists, the case was defined as postoperative recurrence; otherwise, it was defined as non-recurrence. According to early recurrence status, the training cohort was divided into a recurrence group (52 cases) and a non-recurrence group (208 cases).

### 2.3 Statistical analysis

Statistical analyses were performed using SPSS software (version 25.0) and R software (version 4.5.1). All continuous variables were tested using the Kolmogorov–Smirnov test, and continuous variables that followed a normal distribution were expressed as the mean ± standard deviation and compared between groups using the independent-samples *t*-test; continuous variables that did not follow a normal distribution were expressed as M (Q1, Q3) and compared using the Mann–Whitney *U*-test. Categorical variables were expressed as [n (%)] and compared using the *χ*^2^ test. Continuous variables, including the Lund-Mackay score, EOS, and IgE, were analyzed using restricted cubic spline analysis, which showed no evidence of a significant departure from linearity; therefore, these variables were included in the model as linear terms.Risk factors were screened using Lasso regression and logistic regression analyses to identify independent factors influencing early postoperative polyp recurrence in patients with CRSwNP. The “RMS” package was used to construct the Nomogram. The performance and accuracy of the Nomogram model were evaluated using the receiver operating characteristic (ROC) curve, area under the curve (AUC), calibration curve, and the Hosmer–Lemeshow (HL) goodness-of-fit test. Decision curve analysis was applied to assess the clinical utility and net benefit of the Nomogram model. A *P*-value < 0.05 was considered statistically significant.

## 3 Results

### 3.1 Univariate analysis of early postoperative polyp recurrence in CRSwNP patients in the training cohort

There were no statistically significant differences between the non-recurrence group and the recurrence group in terms of sex, alcohol consumption, BMI, number of polyps, hypertension, diabetes mellitus, nasal septal deviation, middle turbinate resection, postoperative nasal adhesion, postoperative nasal packing, long-term use of nasal decongestants, CRP, WBC, PLT, ECP, and IL-1β (*P* > 0.05). The recurrence group showed higher age, longer disease duration, higher Lund–Mackay scores, higher EOS, IgE, and IL-5 levels, as well as higher proportions of smoking, allergic rhinitis, asthma, stage 3 sinusitis, and postoperative infection compared with the non-recurrence group (*P* < 0.05). See Table 1.

**Table 1:** Univariate analysis of early postoperative polyp recurrence in CRSwNP patients using training cohort[n (%) / ( $\bar{x}\pm s$ )/*M*(*Q*_1_*,Q*_3_)].

Indicator	Non recurrence group (*n* = 208)	Recurrence group (*n* = 52)	*χ^2^/t/Z*	*P*
Age (years)	45.36 ± 8.61	50.62 ± 7.54	4.034	＜0.001
Sex [*n*（%）]			1.700	0.192
Male	95（45.67）	29（55.77）		
Female	113（54.33）	23（44.23）		
Smoking [*n*（%）]	72（34.62）	26（50.00）	4.192	0.041
Drinking [*n*（%）]	21（10.10）	9（17.31）	2.120	0.145
BMI（kg/m^2^）	22.91 ± 2.37	22.45 ± 2.92	1.192	0.234
Disease duration (years)	4.72 ± 0.90	5.06 ± 1.14	2.303	0.022
Number of polyps [*n*（%）]			0.198	0.657
Single	83（39.90）	19（36.54）		
Multiple	125（60.10）	33（63.46）		
Hypertension [*n*（%）]	48（23.08）	15（28.85）	0.754	0.385
Diabetes [*n*（%）]	23（11.06）	7（13.46）	0.236	0.627
Allergic rhinitis [*n*（%）]	68（32.69）	25（48.08）	4.286	0.038
Asthma [*n*（%）]	18（8.65）	16（30.77）	17.900	＜0.001
Stages of sinusitis [*n*（%）]			28.117	＜0.001
Stage 1	121（58.17）	12（23.08）		
Stage 2	48（23.08）	13（25.00）		
Stage 3	39（18.75）	27（51.92）		
Deviated nasal septum [*n*（%）]	91（43.75）	25（48.08）	0.315	0.575
Middle turbinate resection [*n*（%）]	50（24.04）	16（30.77）	0.995	0.319
Postoperative infection [*n*（%）]	9（4.33）	7（13.46）	4.533	0.033
Postoperative nasal adhesion [*n*（%）]	22（10.58）	9（17.31）	1.795	0.180
Postoperative tamponade [*n*（%）]	47（22.60）	18（34.62）	3.205	0.073
Long term use of nasal decongestants [*n*（%）]	125（60.10）	33（63.46）	0.198	0.657
Lund-Mackay score（point）	13（10, 16）	16（13, 19）	4.628	＜0.001
CRP（mg/L）	6.5（5.0, 9.6）	7.1（4.8, 10.2）	1.975	0.139
WBC（×10^9^/L）	7.20 ± 2.03	7.81 ± 2.24	1.898	0.059
PLT（×10^9^/L）	286.54 ± 62.61	299.16 ± 68.25	1.277	0.203
EOS（%）	4.25 ± 1.95	6.52 ± 2.04	7.439	＜0.001
ECP（*μ*g/L）	17.5（12.0, 23.7）	19.0（13.1, 25.6）	2.082	0.336
IgE（IU/mL）	180.4（120.6, 262.7）	250.9（194.8, 346.1）	6.176	＜0.001
IL-1β（pg/mL）	11.78 ± 3.26	12.56 ± 4.05	1.466	0.144
IL-5（pg/mL）	12.8（9.6, 16.7）	14.0（10.9,17.8）	3.104	0.008

BMI is body mass index; CRP is C-reactive protein; WBC is white blood cell count; PLT is platelet count; EOS is eosinophil percentage; ECP is eosinophil cationic protein; IgE is immunoglobulin E; IL-1β is interleukin-1β; IL-5 is interleukin-5; *S* is standard deviation.

### 3.2 Lasso regression analysis for screening risk factors of early postoperative polyp recurrence in CRSwNP patients

**Figure 2:**
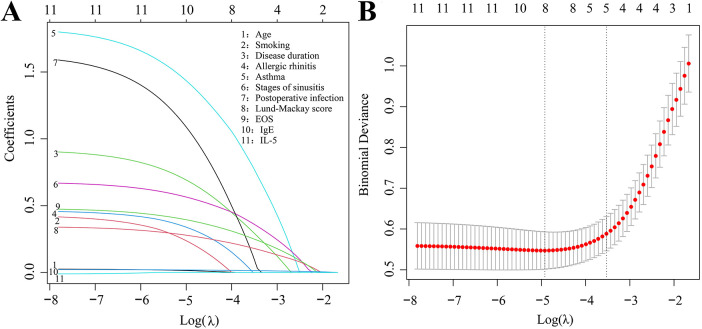
Lasso regression analysis for variable selection **A**: lasso coefficient path plot; **B**: cross-validation curve of lasso regression analysis.

To address multicollinearity, the 11 factors with *P* < 0.05 in the univariate analysis in Section 2.1 were included in Lasso regression, and Lasso regression was applied to select the optimal *λ* and log(*λ*), reducing the number of predictive variables from 11 to 5. These five factors were asthma, sinusitis staging, Lund–Mackay score, EOS, and IgE. Figure 2A presents the Lasso coefficient profiles for variable selection based on the minimum standard error criterion (1se), identifying five variables with non-zero coefficients. Figure 2B illustrates the relationship between deviance (binomial deviance) and log(*λ*), showing that the minimum value across all *λ* values was 1se, corresponding to the minimal standard value of five, which was used as the criterion for selecting optimal non-zero coefficient variables.

### 3.3 Logistic regression analysis for identifying independent factors affecting early postoperative polyp recurrence in CRSwNP patients

Based on the results of the Lasso regression analysis, the five variables with *P* < 0.05 in the Lasso regression analysis were included in a logistic regression analysis, with variable assignments shown in Table 2. The logistic regression analysis is shown in Table 3, and the *β* values, OR values, and other parameters were all derived from the logistic regression model.The results indicated that asthma, sinusitis staging, Lund–Mackay score, EOS, and IgE were all significant independent factors influencing early postoperative polyp recurrence in CRSwNP patients (*P* < 0.05).

**Table 2:** Logistic regression analysis variable assignment table.

Factor	Assignment
Asthma	Yes=1，No = 0
Stages of sinusitis	Stage 3 = 2，Stage 2 = 1，Stage 1 = 0
Lund-Mackay score	Continuous variable
EOS	Continuous variable
IgE	Continuous variable
Dependent variable	Recurrence=1，Non recurrence=0

**Table 3:** Logistic regression analysis of early postoperative polyp recurrence in CRSwNP patients.

Factor	β	SE	Waldχ2	OR	95%CI	*P*
Asthma	1.506	0.613	6.040	4.508	1.357∼14.981	0.014
Stages of sinusitis Stage 1			5.869		0.053	
Stage 2	0.885	0.570	2.407	2.423	0.792∼7.413	0.121
Stage 3	1.248	0.526	5.618	3.482	1.241∼9.769	0.018
Lund-Mackay score	0.331	0.077	18.250	1.392	1.196∼1.621	＜0.001
EOS	0.474	0.125	14.459	1.607	1.258∼2.052	＜0.001
IgE	0.021	0.005	21.875	1.022	1.013∼1.031	＜0.001
Constant	−14.665	2.105	48.536	＜0.001	-	＜0.001

### 3.4 Construction of the nomogram model for early postoperative polyp recurrence in CRSwNP patients

**Figure 3:**
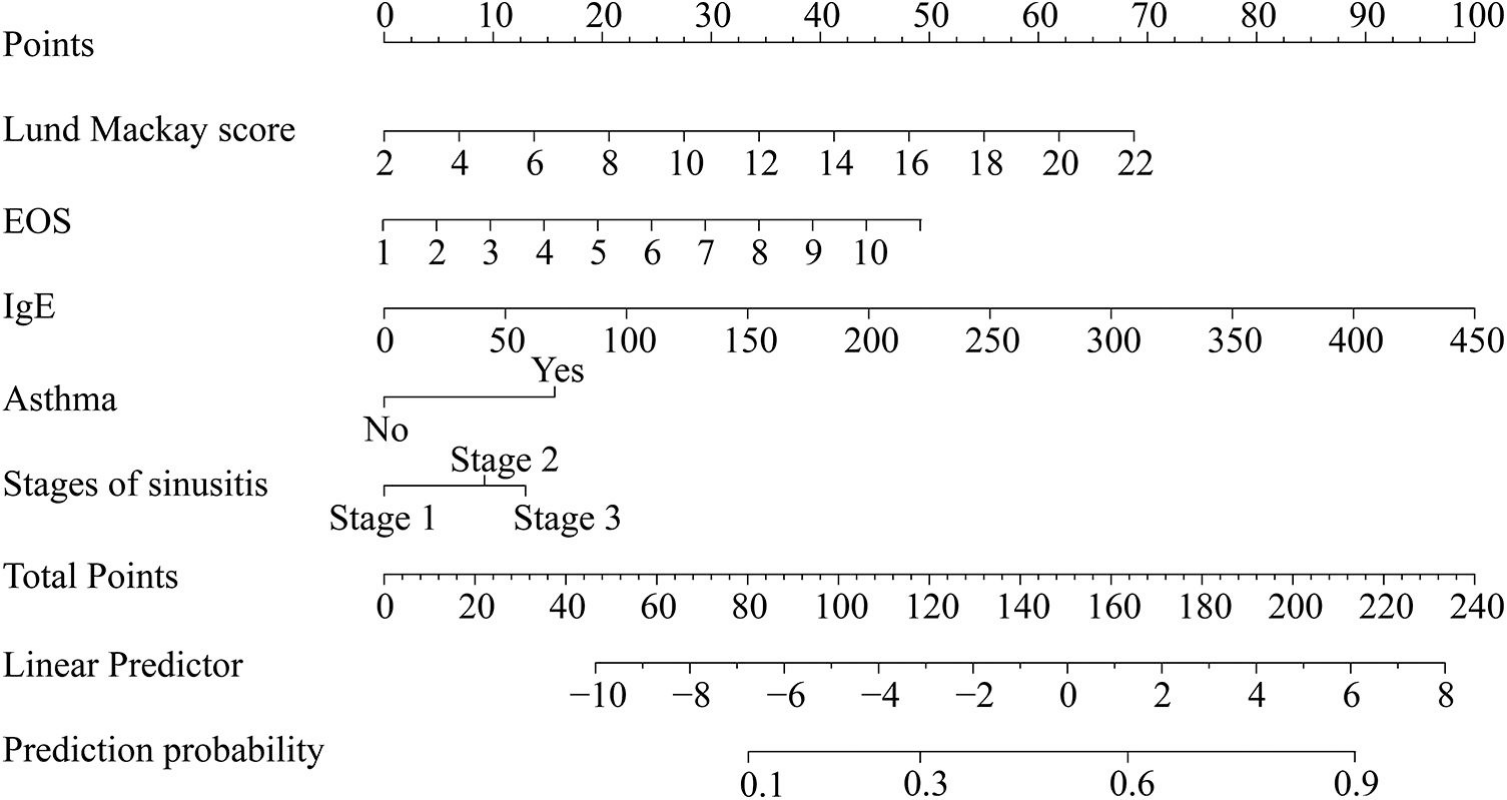
Nomogram prediction model for early postoperative polyp recurrence in CRSwNP patients.

A Nomogram prediction model was constructed using the five independent influencing factors with *P* < 0.05 identified by logistic regression analysis. The model equation based on the *β* coefficients was logit(P) = −14.665 + 1.506 × asthma + 1.248 × sinusitis staging + 0.331 × Lund–Mackay score + 0.474 × EOS + 0.021 × IgE. A Nomogram was developed based on the logistic regression model, as shown in Figure 3. To use the Nomogram, the values of the five variables are first determined, and a vertical line is drawn upward from each value to obtain the corresponding score. The scores for the five variables are then summed to obtain a total score. Next, the total score is located on the “Total Points” axis, and a vertical line is drawn downward from the corresponding point to obtain the predicted probability of early postoperative polyp recurrence in CRSwNP patients.

### 3.5 Validation in the training cohort

**Figure 4:**
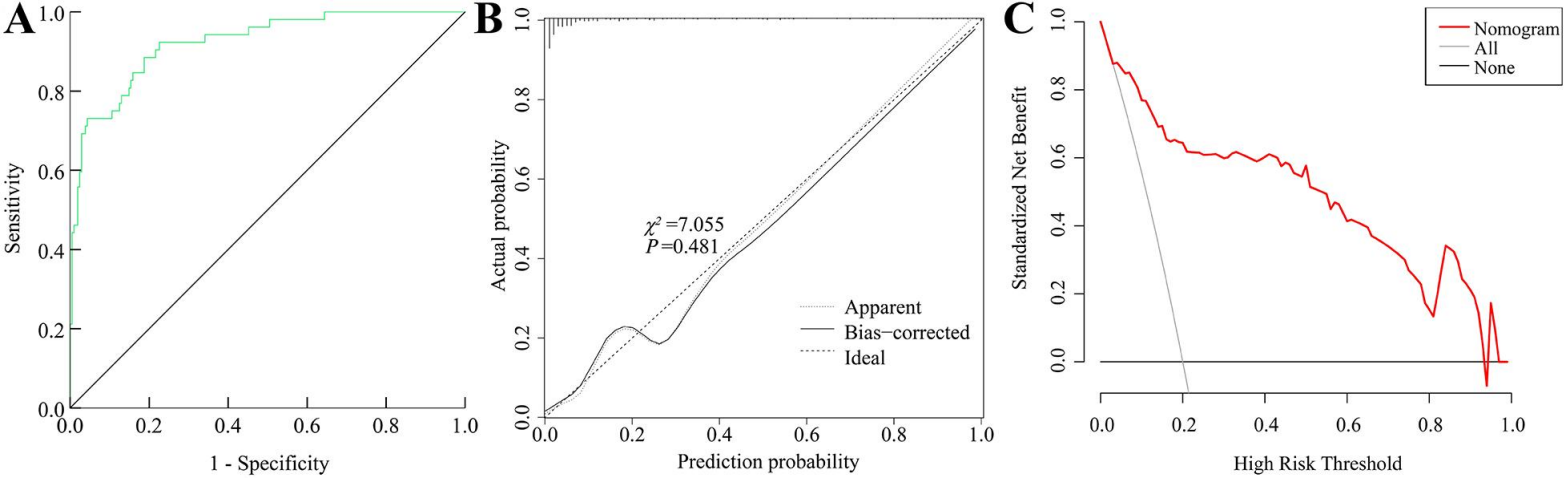
Performance of the nomogram model for predicting early postoperative polyp recurrence in patients with CRSwNP in the training cohort **A**:receiver operating characteristic (ROC) curve; **B**:calibration curve;**C**:decision curve analysis (DCA).

In the training cohort, the AUC (95% CI) of the ROC curve (Figure 4A) for the Nomogram prediction model was 0.923 (0.882–0.963), indicating high discriminative ability. The calibration curve (Figure 4B) demonstrated good agreement between the predicted and observed outcomes, and the HL goodness-of-fit test yielded a *χ*^2^ value of 7.055 (*P* = 0.481), indicating good calibration. The decision curve analysis (Figure 4C) showed that across threshold probabilities ranging from 0.05 to 0.93 at different time points, the net benefit of the Nomogram prediction model was higher than the two extreme strategies of “treat all” and “treat none”.

### 3.6 Validation in the internal validation cohort

**Figure 5:**
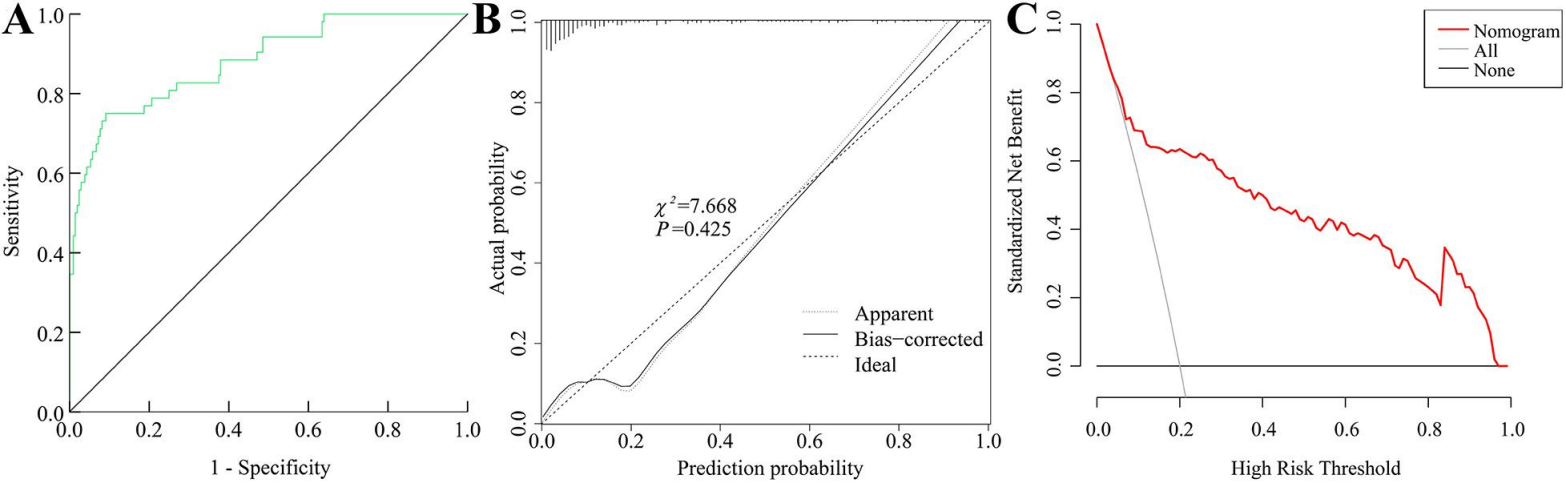
Performance of the nomogram model for predicting early postoperative polyp recurrence in patients with CRSwNP in the internal validation cohort **A**:receiver operating characteristic (ROC) curve;**B**:calibration curve;**C**:decision curve analysis (DCA).

In the internal validation cohort, the AUC (95% CI) of the ROC curve (Figure 5A) for the Nomogram prediction model was 0.881 (0.827–0.936), indicating high discriminative ability. The calibration curve (Figure 5B) showed good agreement between predicted and observed outcomes, and the HL goodness-of-fit test yielded a *χ*^2^ value of 7.668 (*P* = 0.425), indicating good calibration. The decision curve analysis (Figure 5C) demonstrated that across threshold probabilities ranging from 0.09 to 0.97 at different time points, the net benefit of the Nomogram prediction model was higher than the two extreme strategies of “treat all” and “treat none”.

### 3.7 Validation in the external validation cohort

**Figure 6:**
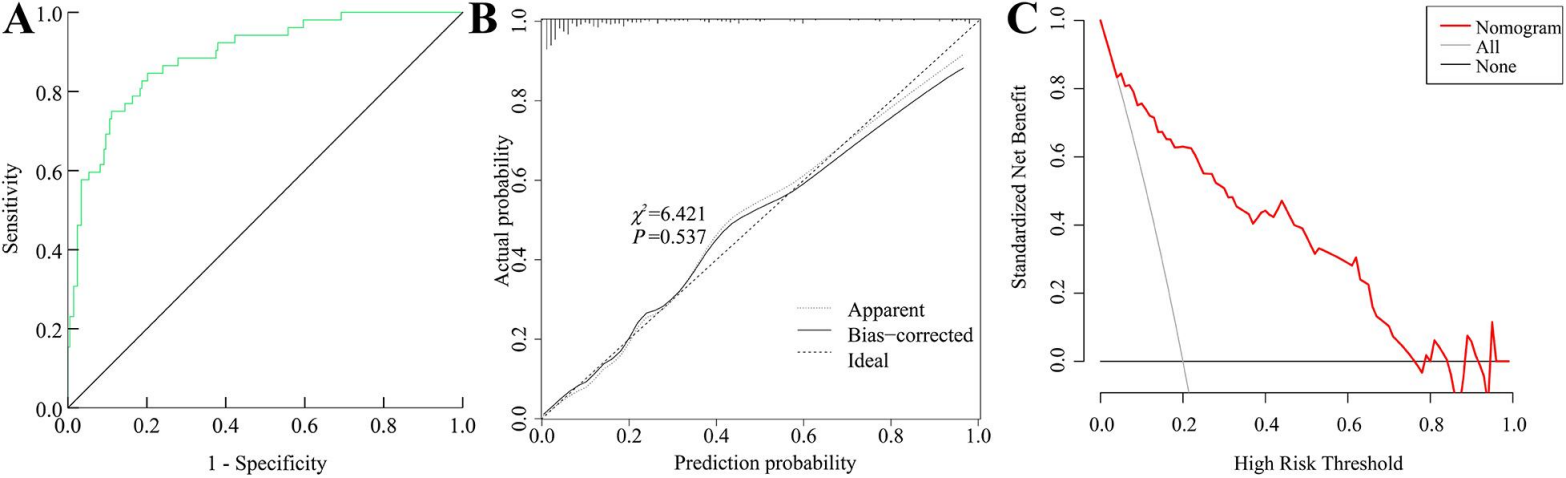
Performance of the nomogram model for predicting early postoperative polyp recurrence in patients with CRSwNP in the external validation cohort **A**:receiver operating characteristic (ROC) curve;**B**:calibration curve;**C**:decision curve analysis (DCA).

In the external validation cohort, the AUC (95% CI) of the ROC curve (Figure 6A) for the Nomogram prediction model was 0.890 (0.841–0.939), indicating high discriminative ability. The calibration curve (Figure 6B) showed good agreement between predicted and observed outcomes, and the HL goodness-of-fit test yielded a *χ*^2^ value of 6.421 (*P* = 0.537), indicating good calibration. The decision curve analysis (Figure 6C) indicated that across threshold probabilities ranging from 0.07 to 0.77 at different time points, the net benefit of the Nomogram prediction model was higher than the two extreme strategies of “treat all” and “treat none”.

## 4 Discussion

The persistently high risk of postoperative polyp recurrence in patients with CRSwNP remains a major challenge in clinical management. How to perform precise stratified intervention has become one of the key focuses of rhinology research in recent years. Based on findings from recent studies, factors such as asthma, sinusitis staging, Lund-Mackay score, EOS, and IgE have gradually been identified as being associated with recurrence. Retrospective analyses by Fageeh et al. (11) and Du et al. (12) both found that asthma was a high-risk clinical feature for recurrence. Previous studies have also reported the effect of sinusitis severity on postoperative recurrence (13, 14). Kirtsreesakul et al. (15) clarified the predictive value of the Lund-Mackay score for recurrence risk. Systematic reviews by Chen et al. (16) and Lin et al. (17) further confirmed the association between type 2 inflammation-related indicators, such as peripheral blood EOS and IgE, and the risk of recurrence. However, existing clinical studies still have limitations. Most studies directly constructed models after screening variables through traditional univariate analysis, without addressing potential multicollinearity among variables, which may easily lead to model overfitting. In addition, some models include overly complicated indicators, making them difficult to apply in routine clinical practice. To address these limitations, this study aimed to construct a simple, accurate, and practical prediction tool for recurrence.

In this study, LASSO regression was used to reduce the dimensionality of the 11 candidate variables screened by univariate analysis. Finally, five factors were selected, including asthma, stage 3 sinusitis, Lund-Mackay score, EOS, and IgE, and the variable selection was statistically reasonable. Among them, asthma was the predictor with the highest weight. Logistic regression showed that asthma increased the risk of recurrence by 4.508 times. This may be because patients with CRSwNP complicated by asthma exhibit similar inflammatory cytokine infiltration responses in the bronchial mucosa and sinonasal mucosa. After surgical removal of nasal mucosal lesions, asthma-related lesions or inflammatory cytokines may migrate to the paranasal sinuses, thereby inducing polyp recurrence (10, 11). Compared with stage 1 sinusitis, stage 3 sinusitis increased the risk of recurrence by 3.482 times. Patients with this type of sinusitis have more severe disease and require more steroid therapy, which may lead to a marked decrease in immune tolerance and increased susceptibility to pathogens. Pathogens may therefore be one of the main causes of disease recurrence (13, 14). The Lund-Mackay score mainly quantifies the severity of sinus lesions by evaluating the degree of opacification of each sinus and obstruction of the ostiomeatal complex on sinus CT images (15, 18, 19). This study further clarified the relationship between the Lund-Mackay score and recurrence. The results confirmed that for each 1-point increase in the score, the risk of postoperative recurrence increased by 1.392 times, reflecting a linear association between the extent of inflammatory involvement and the risk of recurrence. EOS and IgE are core effector molecules of type 2 inflammation (16, 17). Toxic granule proteins released by EOS can directly damage the epithelial barrier of the nasal mucosa and promote tissue fibrosis, while IgE-mediated type I hypersensitivity reactions can continuously recruit inflammatory cell infiltration. Although surgery removes visible polyps, the abnormal immune status may still provide a basis for recurrence (20, 21).

Bai et al. (22) combined tissue biomarkers, including ECP, IL-5, and anti-dsDNA IgG, with clinical variables to develop a risk prediction score, with an AUC of 0.76 in the external validation cohort. Although this score stratified patients with different risks of recurrence, it is too complex for rapid assessment in routine outpatient practice. In contrast, the five factors selected in this study are all commonly used clinical indicators that can be obtained through routine preoperative history-taking, including a history of asthma and sinusitis staging, and routine examinations, including blood routine testing, total IgE, and sinus CT. This indicates that the model does not increase the economic or time burden on patients and does not require special laboratory conditions, making it suitable for rapid assessment and widespread application in outpatient settings at hospitals of different levels. In addition, compared with previous studies, this study introduced LASSO regression for dimensionality reduction during variable selection, which eliminated redundant variables while retaining important variables. This not only improved the stability of the model but also avoided interference from collinearity. Furthermore, this study included data from another hospital for model validation, and the cross-center results confirmed that the model has a certain degree of generalizability.A Nomogram consists of variable names, scales, and assigned values, with each variable corresponding to segmented scales and scores, making it concise, intuitive, and easy to understand and apply (23). By using auxiliary lines and simple summation, a Nomogram allows rapid estimation of the risk of postoperative polyp recurrence in CRSwNP. In terms of model evaluation, previous studies mostly reported only AUC values, whereas this study simultaneously validated discrimination, calibration, and clinical benefit. The AUC reached 0.923 in the training cohort, and the AUCs in the internal validation cohort and external validation cohort were 0.881 and 0.890, respectively, which were significantly higher than the values of 0.738 and 0.853 reported by Xu et al. (24) and 0.80–0.85 reported by Zhao et al. (25).

In addition, in this study, the calibration curves closely approximated the ideal curve, and decision curve analysis confirmed that the Nomogram model provided a high net benefit across a wide range of threshold probabilities. However, considering clinical practicality and feasibility, a threshold probability of 20% (0.2) is recommended as a clinically meaningful risk threshold for postoperative management of CRSwNP. At this threshold, the model showed a positive net benefit, which was clearly higher than those of the “treat-all” and “treat-none” strategies. In addition, approximately 52, 23, and 48 patients in the training cohort, internal validation cohort, and external validation cohort, respectively, were classified as high risk.These findings indicate that the Nomogram developed in this study has certain predictive performance for early postoperative polyp recurrence in CRSwNP patients. Clinicians can use the individual scores of CRSwNP patients to estimate the probability of postoperative recurrence and to identify high-risk patients at an early stage. Furthermore, appropriate interventions targeting controllable risk factors should be implemented to minimize the likelihood of recurrence. The clinical value of this model mainly lies in its ability to rapidly identify high-risk patients using the above five indicators. For patients at high risk of recurrence, the surgical strategy may be adjusted when necessary, including appropriately expanding the extent of sinus ostium opening, simultaneously treating concomitant lesions, strengthening postoperative management, shortening follow-up intervals, and initiating intensive interventions in advance.

This study presents a Nomogram model based on five clinically accessible factors, which, once validated in larger clinical cohorts, has the potential to substantially improve the prediction of early postoperative polyp recurrence in CRSwNP. Despite its promising potential, several limitations must be acknowledged. First, as a retrospective study, there may be selection bias. Second, although this was not a single-center study, the sample size remains relatively small. Third, the number of included influencing factors was limited, and some confounding factors may not have been fully excluded. Fourth, although a Nomogram model was constructed, the underlying biological mechanisms were not fully explored. Fifth, the follow-up period was relatively short, with a follow-up duration of only 6 months. In future studies, the follow-up period will be extended to explore the impact on long-term prognosis. Sixth, this study failed to analyze histological characteristics and local biomarkers.Future prospective studies with larger sample sizes are needed to further validate the stability and generalizability of the model.

In summary, this study successfully developed and evaluated a Nomogram model for predicting early postoperative polyp recurrence in CRSwNP. Five risk factors were identified through Lasso and logistic regression analyses, and the Nomogram demonstrated favorable performance in ROC curve analysis, AUC, calibration curves, HL goodness-of-fit testing, and decision curve analysis. This Nomogram is expected to be useful in clinical practice for predicting early recurrence risk and improving patient prognosis.

### 4.1 Research involving human participants

This retrospective study involving human participants was reviewed and approved by the Medical Ethics Committee of Wenzhou Central Hospital (Approval No. L2026-01-017; approval date: January 23, 2026). The use of the external validation dataset from Wenzhou People's Hospital was reviewed and approved by the Ethics Committee of Wenzhou People's Hospital (Approval No. KY-202603-019; approval date: March 13, 2026). The requirement for written informed consent was waived by the ethics committees because of the retrospective nature of the study and the use of de-identified clinical data. All patient information was de-identified before analysis, and patient confidentiality was strictly protected. The study was conducted in accordance with the ethical principles of the Declaration of Helsinki.

## Data Availability

The original contributions presented in the study are included in the article/Supplementary Material, further inquiries can be directed to the corresponding author/s.
