# Supplementary material for: Development and validation of a nomogram based on LASSO regression for predicting early postoperative polyp recurrence in patients with chronic rhinosinusitis with nasal polyps
Source: Front Surg. 2026 Jul 14;13:1806350. doi: 10.3389/fsurg.2026.1806350 (PMC13407357; doi:10.3389/fsurg.2026.1806350)
Supplement: Supplementary file 1 [file Table1.docx]

**Supplementary Table 1 Baseline characteristics of the training cohort, internal validation cohort, and external validation cohort**

| Indicator | Training cohort（*n*=260） | Internal validation cohort（*n*=114） | External validation cohort（*n*=242） |
| --- | --- | --- | --- |
| Age (years) | 46.41±8.20 | 47.28±8.96 | 45.49±8.75 |
| Sex[*n*（%）] |  |  |  |
| Male | 124（47.69） | 51（44.74） | 124（51.24） |
| Female | 136（52.31） | 63（55.26） | 118（48.76） |
| Smoking[*n*（%）] | 98（37.69） | 48（42.11） | 114（47.11） |
| Drinking[*n*（%）] | 30（11.54） | 20（17.54） | 41（16.94） |
| BMI（kg/m^2^） | 22.82±2.50 | 22.36±2.81 | 22.97±2.35 |
| Disease duration (years) | 4.79±0.95 | 4.86±0.98 | 4.72±0.93 |
| Number of polyps[*n*（%）] |  |  |  |
| Single | 102（39.23） | 41（35.96） | 106（43.80） |
| Multiple | 158（60.77） | 73（64.04） | 136（56.20） |
| Hypertension[*n*（%）] | 63（24.23） | 32（28.07） | 58（23.97） |
| Diabetes[*n*（%）] | 30（11.54） | 11（9.65） | 37（15.29） |
| Allergic rhinitis[*n*（%）] | 93（35.77） | 35（30.70） | 72（29.75） |
| Asthma[*n*（%）] | 34（13.08） | 18（15.79） | 27（11.16） |
| Stages of sinusitis[*n*（%）] |  |  |  |
| Stage 1 | 133（51.15） | 52（45.62） | 129（53.31） |
| Stage 2 | 61（23.46） | 28（24.56） | 51（21.07） |
| Stage 3 | 66（25.39） | 34（29.82） | 62（25.62） |
| Deviated nasal septum[*n*（%）] | 116（44.62） | 48（42.11） | 86（35.54） |
| Middle turbinate resection[*n*（%）] | 66（25.38） | 25（21.93） | 50（20.66） |
| Postoperative infection[*n*（%）] | 16（6.15） | 13（11.40） | 20（8.26） |
| Postoperative nasal adhesion[*n*（%）] | 31（11.92） | 10（8.77） | 26（10.74） |
| Postoperative tamponade[*n*（%）] | 65（25.00） | 22（19.30） | 51（21.07） |
| Long term use of nasal decongestants[*n*（%）] | 158（60.77） | 62（54.39） | 138（57.02） |
| Lund-Mackay score（point） | 13（11，18） | 13（10，16） | 13（11，17） |
| CRP（mg/L） | 6.6（5.1，9.9） | 6.9（5.3，9.2） | 7.0（5.6，9.2） |
| WBC（×10^9^/L） | 7.32±2.10 | 7.18±2.33 | 7.46±2.09 |
| PLT（×10^9^/L） | 289.07±63.91 | 294.33±71.65 | 298.62±59.24 |
| EOS（%） | 4.70±1.98 | 4.25±1.19 | 4.62±1.67 |
| ECP（μg/L） | 17.8（13.0，24.6） | 18.3（14.1，23.5） | 17.5（12.4，22.0） |
| IgE（IU/mL） | 198.5（132.4，309.8） | 203.1（155.0，316.8） | 212.5（163.7，306.2） |
| IL-1β（pg/mL） | 11.91±3.49 | 11.22±3.62 | 12.04±3.25 |
| IL-5（pg/mL） | 12.5（9.8，17.4） | 12.3（9.7，15.1） | 12.5（10.0，15.8） |

Note: Data are presented as mean ± standard deviation, median (interquartile range), or n (%), as appropriate. BMI, body mass index; CRP, C-reactive protein; WBC, white blood cell count; PLT, platelet count; EOS, eosinophil percentage; ECP, eosinophil cationic protein; IgE, immunoglobulin E; IL-1β, interleukin-1β; IL-5, interleukin-5.
